# P-Doped Carbon Quantum Dots with Antibacterial Activity

**DOI:** 10.3390/mi12091116

**Published:** 2021-09-16

**Authors:** Shuiqin Chai, Lijia Zhou, Shuchen Pei, Zhiyuan Zhu, Bin Chen

**Affiliations:** 1 Chongqing Key Laboratory of Industrial Fermentation Microorganism, Chongqing University of Science and Technology, No. 20 East Daxuecheng Road, Chongqing 401331, China; peishuchen928@163.com; 2 College of Chemistry and Chemical Engineering, Chongqing University of Science and Technology, No. 20 East Daxuecheng Road, Chongqing 401331, China; lijiazhou1128@163.com; 3 Chongqing Key Laboratory of Non-Linear Circuit and Intelligent Information Processing, College of Electronic and Information Engineering, Southwest University, Chongqing 400715, China; zyuanzhu@swu.edu.cn; 4 Ocean College, Zhejiang University, Hangzhou 316021, China

**Keywords:** carbon quantum dots, biocompatibility, antibacterial activity

## Abstract

It is a major challenge to effectively inhibit microbial pathogens in the treatment of infectious diseases. Research on the application of nanomaterials as antibacterial agents has evidenced their great potential for the remedy of infectious disease. Among these nanomaterials, carbon quantum dots (CQDs) have attracted much attention owing to their unique optical properties and high biosafety. In this work, P-doped CQDs were prepared by simple hydrothermal treatment of m-aminophenol and phosphoric acid with fluorescence emission at 501 nm when excited at 429 nm. The P-doped CQDs showed effective antibacterial activity against *Escherichia coli* (*E. coli*) and *Staphylococcus aureus* (*S. aureus*). The minimal inhibitory concentrations (MICs) of P-doped CQD were 1.23 mg/mL for *E. coli* and 1.44 mg/mL for *S. aureus*. Furthermore, the morphologies of *E. coli* cells were damaged and *S. aureus* became irregular when treated with the P-doped CQDs. The results of zeta potential analysis demonstrated that the P-doped CQDs inhibit antibacterial activity and destroy the structure of bacteria by electronic interaction. In combination, the results of this study indicate that the as-prepared P-doped CQDs can be a promising candidate for the treatment of bacterial infections.

## 1. Introduction

Bacterial infections, especially those caused by drug-resistant bacteria, threaten public health and have always been a serious problem worldwide [1,2]. Due to the abuse of antibiotics, these infections have become more difficult to cure because of increasingly serious drug-resistance [3,4]. It is of urgent concern to develop alternative antimicrobial agents with excellent properties against bacterial infection. Recently, nanomaterials involving metal or metal oxide have aroused widespread interest due to their great potential in the treatment of bacterial infections from antibiotic-resistant bacteria [5,6,7,8]. However, it is essential to balance antibacterial efficiency and biosafety, which has become an obstacle in the clinical application of these semiconductor nanomaterials. Carbon-based nanomaterials, including fullerene, carbon nanotubes, graphene, graphene oxide, and carbon quantum dots [9,10,11], have been proved to have efficient antimicrobial activity and high biocompatibility. Therefore, these carbon-based nanomaterials offer more potential for the elimination of various bacterial infections with negligible cytotoxicity and excellent biocompatibility.

Carbon quantum dots (CQDs), a new type of zero-dimensional carbon-based nanomaterial, exhibit intriguing properties, such as photostability, great environmental sustainability, high water dispersibility, easy synthesis, low production cost, and low toxicity, as well as good biocompatibility [12,13,14,15]. These characteristics mean that CQDs are excellent candidates for use as optical nanoprobes for chemical analysis [16,17], and for fingerprint imaging [18], biosensors [19,20], fluorescent labeling, and bioimaging [21,22]. Recently, numerous studies on CQDs with antibacterial activity have been reported. Huang et al. reported that super-cationic CQDs possessed strong antibacterial activities against multidrug-resistant bacteria and superior efficacy in treating eye-related bacterial infections [23]. Qu et al. reported that various surface-charged carbon dots induced programmed bacterial death and systematically analyzed the apoptosis mechanisms of *E. coli* cells [24]. Kang et al. developed a series of cationic carbon dots, which can selectively kill Gram-positive bacteria via electrostatic interaction, with a greater positive charge showing stronger the antibacterial ability [25]. Among them, the most positively charged CQDs have shown a considerable capacity for the inhibition of bacterial growth due to their strong antibacterial effect by interacting with the negatively charged components of bacterial cell walls. Therefore, it is critical to regulate the surface charge and to synthesize CQDs with a controllable positive charge.

Inspired by these superior properties and the potential applications, more researchers have focused on the preparation of positively charged CQDs. The heteroatoms, such as nitrogen (N) and boron (B), when used in the doping of CQDs, can effectively tune the electronic energy level and affect certain intrinsic chemical properties of CQDs, which has attracted growing interest from researchers [20,26,27]. The N atom has been widely used for chemical doping in CQDs because of its five valence electrons strongly bonding with carbon atoms [28,29]. Although the phosphorus (P) atom is larger than the carbon atom, it can work as an n-type donor to alter the electronic characteristics of CQDs [30,31]. Thus, in order to create more active electronic sites, the P atom is used as a heteroatom to synthesize the CQDs in our work, which could produce unanticipated and exceptional antibacterial effects.

**Scheme 1:**
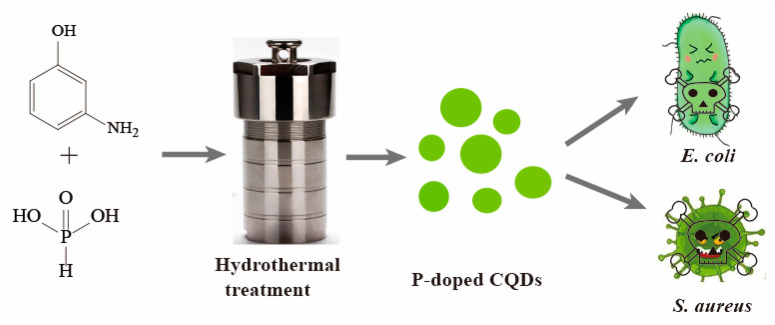
Illustration of the synthesis of P-doped CQDs by one-step hydrothermal treatment for the effective killing of *E. coli* and *S. aureus* bacteria.

In this work, the P-doped CQDs derived from *m*-aminophenol and phosphoric acid were first prepared by one-step hydrothermal treatment. In vitro cytotoxicity analyses revealed the good biocompatibility of P-doped CQDs. Importantly, the as-prepared P-doped CQDs showed excellent inhibitory effects on *Escherichia coli* (*E. coli*, Gram-negative bacteria) and *Staphylococcus aureus* (*S. aureus*, Gram-positive bacteria). Furthermore, the cell walls of the bacteria were broken after P-doped CQD treatment, and the zeta potential of P-doped CQDs was +23.1 mV. Thus, the antibacterial mechanism of P-doped CQDs may be attributed to the electrostatic interaction between the surface charge of P-doped CQDs and bacterial cell walls (Scheme 1).

## 2. Materials and Methods

### 2.1. Materials

*M*-aminophenol (≥98%) was purchased from the Aladdin Reagent Co. Ltd. (Shanghai, China). A Britton–Robinson (BR) buffer and sodium chloride (NaCl) solution were prepared and adjusted to control the acidity and the ionic strength. Ultra-pure water (18.2 MΩ), purified by a Millipore water purification system (Milli-Q, Millipore, Billerica, MA, USA), was used for the preparation of all solutions.

### 2.2. Apparatus

All fluorescence spectra were acquired using a Shimatzu RF-5301PC fluorescence spectrophotometer (Kyoto, Japan). Transmission electron microscopy (TEM) and high-resolution transmission electron microscopy (HRTEM) images were performed on a JEOL 2100F field emission transmission electron microscope (Tokyo, Japan). The thickness of CQDs was characterized using a Dimension Icon Scan Asyst atomic force microscope (AFM, Bruker Co., Karlsruhe, Germany). Elemental and functional group analyses were performed using an ESCALAB Xi^+^ X-ray photoelectron spectrometer (XPS, Thermo Fisher Scientific Inc., Waltham, MA, USA) and a Nicolet iS5 Fourier Transform Infrared spectrometer (FTIR, Thermo Fisher Scientific Inc., Waltham, MA, USA). The Raman spectrum obtained on the Ag substrate (excited by a 532 nm laser) was recorded with a DXR 2xi Raman microscope (Thermo Fisher Scientific Inc., Waltham, MA, USA). The C9920-02G fluorescence spectrophotometer (Hamamatsu Photonics KK, Tokyo, Japan) was used to measure the fluorescence lifetime. Zeta potentials of CQDs were obtained with a Zetasizer Nano ZS90 System (Malvern, UK). The concentration of bacteria was determined by measuring the optical density at 600 nm (OD_600_) via UV–vis spectroscopy. The morphology of bacteria was observed under a Hitachi S-3400N scanning electron microscope (SEM, Tokyo, Japan).

### 2.3. Preparation of the P-Doped CQDs

The P-doped CQDs were prepared by one-step hydrothermal treatment using m-aminophenol and phosphoric acid. Briefly, 0.10 g m-aminophenol and 1 ml phosphoric acid was first dispersed in 4.0 mL ultrapure water, and 5.0 mL of the mixture was added into a 25 mL teflon-lined autoclave and then kept at 200 °C for 48 h. Once cooled to room temperature naturally, the resultants were centrifuged at 6000 rpm for 5 min and then the supernatant was filtered with BIOSHARP membrane filters (0.22 μm) to remove insoluble impurities, and the transparent solution was subjected to dialysis (Mw = 500 Da) for 48 h to eliminate the residual unreacted material. Finally, the purified CQD solution was freeze-dried to obtain solid powder. The product was stored at 4 °C for future experiments.

### 2.4. Cellular Toxicity Test

In Dulbecco’s Modified Eagle’s Medium (DMEM) supplemented with 10% fetal bovine serum (FBS), 1 × 10^5^ cells per mL BV2 microglia cells were plated on a 96-well cell culture plate (100 μL per well) and cultured at 37 °C with 5.0% CO_2_ in a humidified incubator for 24 h. The medium was then replaced with 100 μL of DMEM medium supplemented with 2% FBS containing different doses of P-doped CQDs and was incubated for another 24 h. The control was cells without treatment with the P-doped CQDs. After removing the culture medium, a mixture of 10 μL MTT reagent and 90 μL DMEM medium was added to every well, which was washed with PBS buffer three times. The cells were further incubated for 1 h. After removing the culture medium with MTT, 150 μL of DMSO was added and shaken at room temperature for approximately 10 min. The OD was measured at 538 nm. The cell viability was estimated using Equation (1):

Cell viability [%] = (OD_treated_/OD_control_) × 100% (1)

where OD_treated_ and OD_control_ were the optical density of cells in the presence and absence of CQDs, respectively.

### 2.5. MIC Test

*E. coli* and *S. aureus* were cultured in Luria–Bertan (LB) liquid medium at 37 °C shaking at 180 rpm overnight. Bacteria were diluted to a concentration of 1.5 × 10^7^ CFU/mL. A measure of 50 μL of diluted bacterial suspension and 50 μL of various concentrations of P-doped CQDs were transferred to a 96-well cell culture plate for a final volume of 100 μL/well. Bacterial suspension in an LB medium without the P-doped CQDs was used as the control, and only LB medium was used as the blank. The mixtures were incubated at 37 °C for 12 h. At the end of incubation, the concentration of bacteria was determined by measuring OD_600_.

### 2.6. SEM Images for Bacteria

*E. coli* and *S. aureus* were incubated with the as-prepared P-doped CQDs for 4 h. Bacteria without the P-doped CQDs treatment were used as the control groups. The cells were centrifuged at 8000 rpm for 10 min at 4 °C and the precipitation was washed with phosphate-buffered solution (PBS, pH = 7.4). The cells were fixed with 5% glutaraldehyde for 2 h at 4 °C. The bacteria were soaked in a dehydration solution with different concentrations of alcohol (30%, 50%, 70%, 85%, 95%, 100%), which were dried by vacuum and sputter-coated with gold. The samples were observed under a Hitachi S-3400N scanning electron microscope.

## 3. Results and Discussion

### 3.1. Characterizations of the P-Doped CQDs

**Figure 1:**
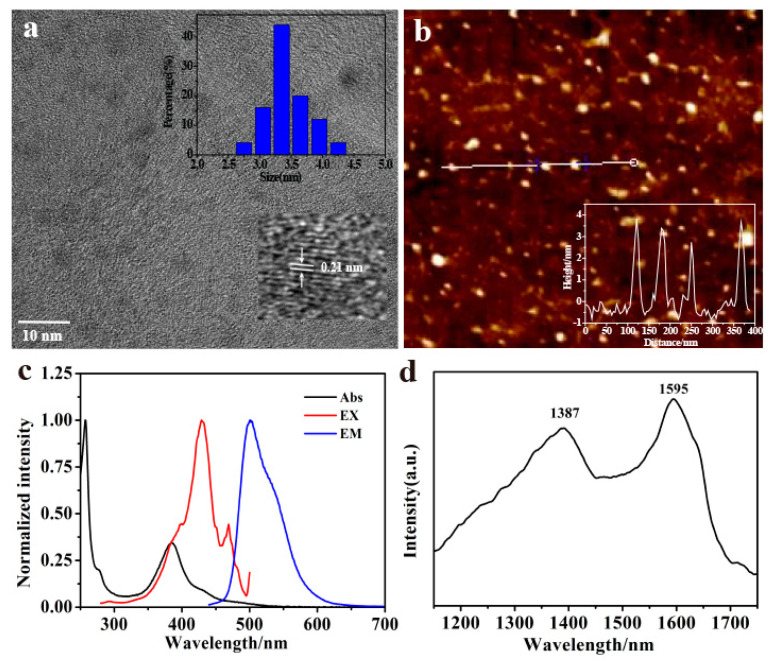
(**a**) TEM images (inset: particle size distribution and HRTEM images). (**b**) AFM images (inset: the height profile). (**c**) Normalized UV-vis absorption spectra, fluorescent excitation, and emission spectra of P-doped CQDs. (**d**) The Raman spectra of P-doped CQDs.

The TEM image (Figure 1a) shows that the size of P-doped CQDs was distributed in the range from 2.75 nm to 4.25 nm with an average diameter of 3.4 nm. The HRTEM image (inset of Figure 1a) shows 0.21 nm of in-plane lattice spacings ([100] facet), revealing a typical graphite-like structure formed during the synthesis of P-doped CQDs [25,32]. The AFM image (Figure 1b) shows that the topographic height of the P-doped CQDs varied mostly from 2.7 nm to 3.8 nm, which was similar to the TEM characterization, indicating that the P-doped CQDs had a nearly spherical morphology.

The P-doped CQDs had unique absorption and emission abilities. As shown in Figure 1c, the P-doped CQDs had a strong absorption band at 257 nm and a broad absorption band characterized at 384 nm owing to the *π→π* * transition of C=C and the *n→π* * transition of the surface groups, respectively. The maximum emission peak was located at 501 nm with a maximum excitation at 429 nm. The fluorescence emission of P-doped CQDs was independent with the excitation wavelength ranging from 300 nm to 460 nm (Figure S1a). In addition, the absolute quantum yield (QY) of P-doped CQDs was approximately 14.4%. The average fluorescence lifetime of P-doped CQDs was calculated as 3.9 ns (Figure S1b).

**Figure 2:**
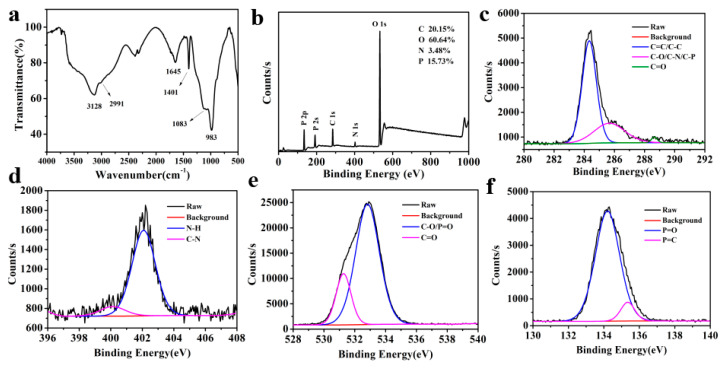
(**a**) The FTIR spectra of P-doped CQDs. (**b**) The XPS spectra of P-doped CQDs. High resolution XPS spectra of P-doped CQDs: (**c**) C 1s, (**d**) N 1s, (**e**) O 1s, and (**f**) P 2p spectra.

In order to understand the functional group composition and structures of P-doped CQDs, Raman spectra, FTIR spectra, and XPS were measured. The Raman spectrum (Figure 1d) illustrated two narrow peaks, a G-line peak at around 1595 cm^−1^ and a D-line peak at around 1387 cm^−1^, which was attributed, respectively, to sp^2^ hybridized carbon and sp^3^ hybridized carbon in the P-doped CQDs. The FTIR spectra (Figure 2a) of P-doped CQDs showed the characteristic absorption bands of O-H and N-H vibrations around 3128 cm^−1^ (which improve the hydrophilicity and stability in the aqueous solution), C-H stretching vibrations at 2991 cm^−1^, C=O bond stretching vibrations around 1645 cm^−1^, C-N bond stretching vibrations around 1401 cm^−1^, C-O stretching vibrations at 1083 cm^−1^, and P-O stretching vibrations at 958 cm^−1^ [16,26,33]. The FTIR spectrum revealed that the P-doped CQDs had abundant carboxyl, hydroxy, and amino functional groups, and that P had indeed been doped into the P-doped CQDs in the synthesis process. The XPS full spectrum of P-doped CQDs (Figure 2b) presented five dominant peaks of C 1s at 283.7 eV, O 1s at 533.5 eV, N 1s at 401.3 eV, and P 2s and P 2p at 191.5 eV and 133.5 eV, respectively, further indicating the successful incorporation of the P element into the P-doped CQDs [26,30]. The elemental analysis showed the composition of C 20.15%, O 60.64%, N 3.48%, and P 15.72%, in the as-prepared P-doped CQDs. The high-resolution C 1s spectra of P-doped CQDs (Figure 2c) was divided into three peaks at 284.3, 285.7, and 288.6 eV, corresponding to C=C/C–C, C-O/C-N/C-P, and C=O, respectively [20,34]. High-resolution N 1s spectra of P-doped CQDs (Figure 2d) showed two peaks at 400.1 and 402.1 eV, which were assigned to pyrrolic N (C-N) and N-H bands, respectively [32]. The bands in the O 1s spectra (Figure 2e) presented two peaks at 531.3 and 532.8 eV, attributed to C=O and C-O/P=O, respectively [35]. The high-resolution P 2p spectra (Figure 2f) revealed the existence of P=O (134.2 eV) and P=C (135.3 eV) [36]. As shown by the results of FTIR and XPS, the as-prepared P-doped CQDs had abundant functional groups, including -COOH, -OH, and a small number of N, P-containing groups.

### 3.2. Stability of the As-Prepared P-Doped CQDs

In order to determine the stability of P-doped CQDs, a series of experiments were designed. The P-doped CQDs showed excellent photostability under continuous Xe lamp illumination (429 nm) for 60 min (Figure S2a). The fluorescence intensity of P-doped CQDs was pH-independent in the BR pH range 2.09–11.92 (Figure S2b). The fluorescence retained 78% of the initial intensity after incubation with 2.0 M NaCl (Figure S2c), illustrating that the P-doped CQDs were stable in a medium of high ionic strength and not aggregated in this medium. The fluorescence intensity of P-doped CQDs slightly decreased when the concentrations of H_2_O_2_ were as high as 500 mM (Figure S2d), indicating that the P-doped CQDs had good antioxidation. These results indicate that the as-prepared P-doped CQDs have great potential in the application of fluorescent nanoprobes for complex matrixes.

### 3.3. Cellular Toxicity and Confocal Microscopy Imaging of the P-Doped CQDs

**Figure 3:**
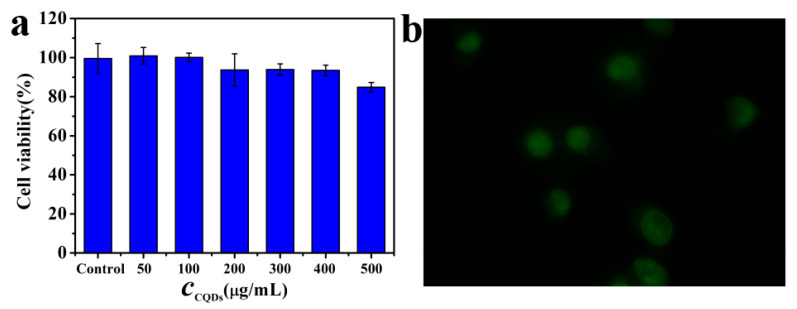
(**a**) Cellular toxicity of P-doped CQDs on BV2 microglioma cell viability. (**b**) Cells imaged by excitation at the WIBA channel.

The cytotoxicity of P-doped CQDs was evaluated by the MTT method using BV2 microglioma cells. The cell viability changed slightly after 24 h incubation with a relatively high concentration of P-doped CQDs (500 µg/mL) (Figure 3a), suggesting that the CQDs had good biocompatibility. To explore the potential application of P-doped CQDs in bioimaging, cellular imaging was investigated through a confocal fluorescence microscope. The confocal image showed that the P-doped CQDs were dispersed in the cytoplasmic and nuclear area (Figure 3b), indicating that the P-doped CQDs can be applied for cytoplasmic and nuclear staining and labelling.

### 3.4. Antibacterial Activity of the P-Doped CQDs

**Figure 4:**
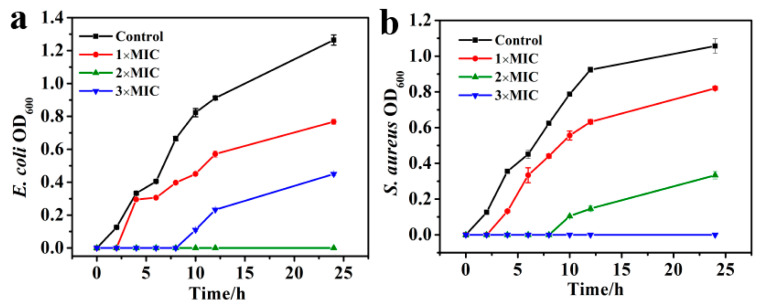
Inhibition effect of P-doped CQDs on the growth of (**a**) *E. coli* and (**b**) *S. aureus*.

The antibacterial activity of P-doped CQDs was explored with *E. coli* and *S. aureus*, as the model pathogens of Gram-negative and Gram-positive bacteria, respectively. Equal amounts of 1.5 × 10^7^ CFU/mL bacterial suspension were treated with different concentrations (0, 0.41, 0.82, 1.23, 1.44, 1.64, 1.84, 2.05 mg/mL) of P-doped CQDs. The bacterial viability was evaluated by recording OD_600_ values of the bacterial mixture. The results showed that the viability of *E. coli* and *S. aureus* decreased with the increasing concentration of P-doped CQDs (Figure S3a,b; Tables S1 and S2). When the concentration of P-doped CQDs was up to 1.23 and 1.44 mg/mL, respectively, the OD_600_ values were almost consistent with that of the blank group, indicating that the P-doped CQDs had significant antibacterial abilities with MIC values of P-doped CQDs of 1.23 mg/mL on *E. coli* and 1.44 mg/mL on *S. aureus*. Furthermore, the P-doped CQDs could effectively inhibit Gram-negative and Gram-positive bacteria on a time-dependent basis at different doses (Figure 4a,b).

**Figure 5:**
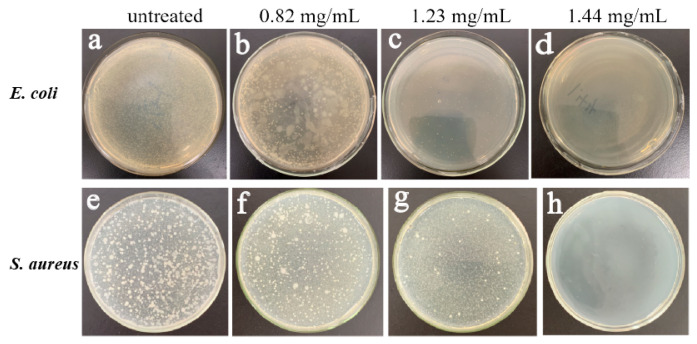
Photographs of culture dishes containing *E. coli* (**a**–**d**) and *S. aureus* (**e**–**h**) treated with 0, 0.82, 1.23, and 1.44 mg/mL, respectively.

In addition, different concentrations of P-doped CQDs (0, 0.82, 1.23, 1.44 mg/mL) were added into the bacterial LB medium to form the LB plate. After incubation for 12 h, bacterial colonies were generated on the LB culture plate. The visual evidence of the antibacterial effect was displayed on the culture plate. The viability of *E. coli* and *S. aureus* showed a concentration-dependent inhibitory effect (Figure 5a–h), similar to the results of the MIC experiments.

### 3.5. Antibacterial Mechanism of P-Doped CQDs

**Figure 6:**
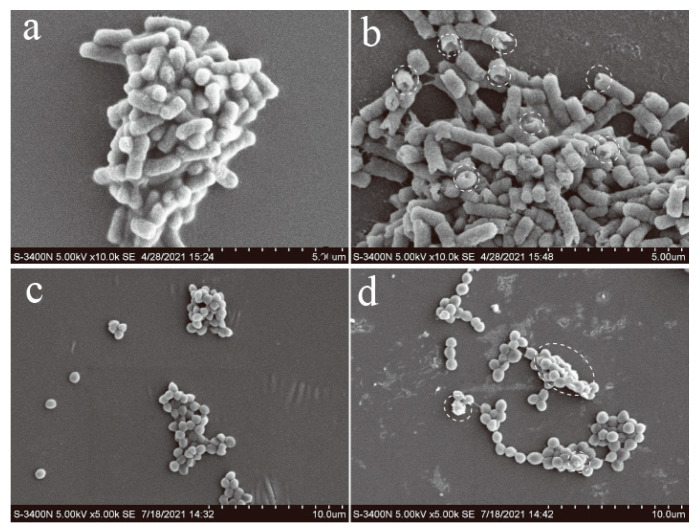
SEM images of: (**a**) *E. coli* without the P-doped CQDs, (**b**) with 1.23 mg/mL of P-doped CQDs, (**c**) *S. aureus* without the P-doped CQDs, (**d**) with 1.44 mg/mL of P-doped CQDs.

**Figure 7:**
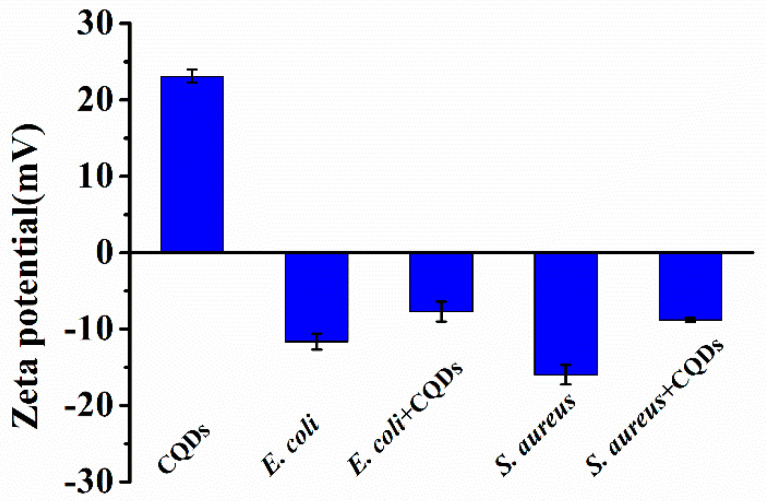
Zeta potentials of P-doped CQDs for *E. coli* and *S. aureus* without and with the P-doped CQDs treatment.

To further explore the mechanism, the morphologies of bacteria, before and after P-doped CQDs treatment, were observed by SEM. *E. coli* and *S. aureus* were incubated with the P-doped CQDs (1.23 mg/mL and 1.44 mg/mL) for 4 h. It was obvious that the integrity of *E. coli* without the P-doped CQDs treatment was kept well, while the cell walls of *E. coli* with the P-doped CQDs treatment were wrinkled and broken (Figure 6a,b). Similarly, the cell walls of *S. aureus* incubated without the P-doped CQDs were intact and smooth, while the cell surfaces of *S. aureus* incubated with the P-doped CQDs became damaged, with some cytoplasm exudated from the cells (Figure 6c,d). Furthermore, zeta potential analysis (Figure 7) showed that the P-doped CQDs were positively charged (+23.1 mV), and *E. coli* (−11.6 mV) and *S. aureus* (−16.0 mV) were negatively charged. The zeta potential was slightly more positive (−7.7 mV for *E. coli* and −8.8 mV for *S. aureus*) after the bacteria were treated with the P-doped CQDs, indicating that the positively charged P-doped CQDs were likely to be bonded with the bacteria by electrostatic interaction, ultimately resulting in the cell walls being disturbed and the death of the bacteria.

## 4. Conclusions

In summary, we have prepared P-doped CQDs with *m*-aminophenol as a carbon source and phosphoric acid as a phosphorus source by a facile hydrothermal method. Based on surface functional group analyses of the as-prepared P-doped CQDs by FTIR and XPS, the P-doped CQDs were successfully doped with phosphorus. The as-prepared P-doped CQDs have high photostability and environmental stability. Furthermore, the P-doped CQDs exhibit a strong inhibitory effect on Gram-negative bacteria (*E. coli*) and Gram-positive bacteria (*S. aureus*) with MIC at 1.23 mg/mL and 1.44 mg/mL, respectively. The Zeta potential measurements indicate that a strong electrostatic interaction between the negatively charged bacteria and the positively charged P-doped CQDs causes the wrinkled and damaged bacteria. This work is of great significance for the development of antibacterial nanomaterials as promising alternative antibacterial agents.

## Supplementary Materials

The following are available online at www.mdpi.com/article/10.3390/mi12091116/s1, Figure S1. (a) Fluorescent emission spectra of P-doped CQDs under different excitation wavelengths from 300 to 460 nm, (b) fluorescence lifetime of P-doped CQDs; Figure S2. Fluorescent spectra of P-doped CQDs at (a) different incubation times, (b) pH solutions, (c) different concentration of NaCl, (d) H_2_O_2_; Figure S3: The antibacterial ability of P-doped CQDs on (a) *E. coli* and (b) *S. aureus.* Table S1: OD values of *E. coli* treated with different concentrations of P-doped CQDs. Table S2: OD values of *S. aureus* treated with different concentrations of P-doped CQDs.
