# Supplementary material for: P-Doped Carbon Quantum Dots with Antibacterial Activity
Source: Micromachines (Basel). 2021 Sep 16;12(9):1116. doi: 10.3390/mi12091116 (PMC8466419; doi:10.3390/mi12091116)
Supplement: Supplementary file 1 [file micromachines-12-01116-s001.zip › micromachines-1374323-supplementary.pdf]

micromachines

Supplementary Materials: P-Doped Carbon Quantum Dots with Antibacterial Activity

Shuiqin Chai [1,2,*], Lijia Zhou [2], Shuchen Pei [1,2], Zhiyuan Zhu [3,4] and Bin Chen [3,*]

1 Chongqing Key Laboratory of Industrial Fermentation Microorganism, Chongqing University of Science and Technology, No. 20 East Daxuecheng Road, Chongqing 401331, China; peishuchen928@163.com
2 College of Chemistry and Chemical Engineering, Chongqing University of Science and Technology, No. 20 East Daxuecheng Road, Chongqing 401331, China; lijiazhou1128@163.com
3 Chongqing Key Laboratory of Non-linear Circuit and Intelligent Information Processing, College of Electronic and Information Engineering, Southwest University, Chongqing 400715,China; zyuanzhu@swu.edu.cn
4 Ocean College, Zhejiang University, Hangzhou 316021, China
* Correspondence: chai0104sq@126.com (S.C.); chenbin121@swu.edu.cn (B.C.); Tel.: +86-23-6825-0394 (B.C.)

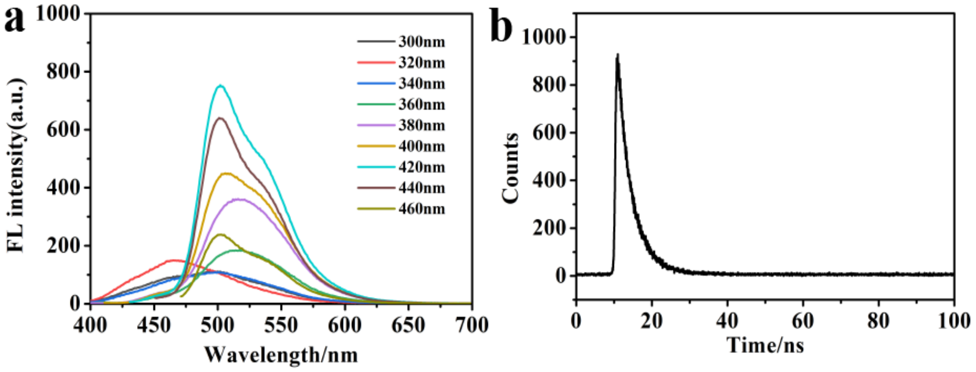

Figure S1. (**a**) Fluorescent emission spectra of P-doped CQDs under different excitation wavelengths from 300 to 460 nm. (**b**) Fluorescence lifetime of P-doped CQDs.

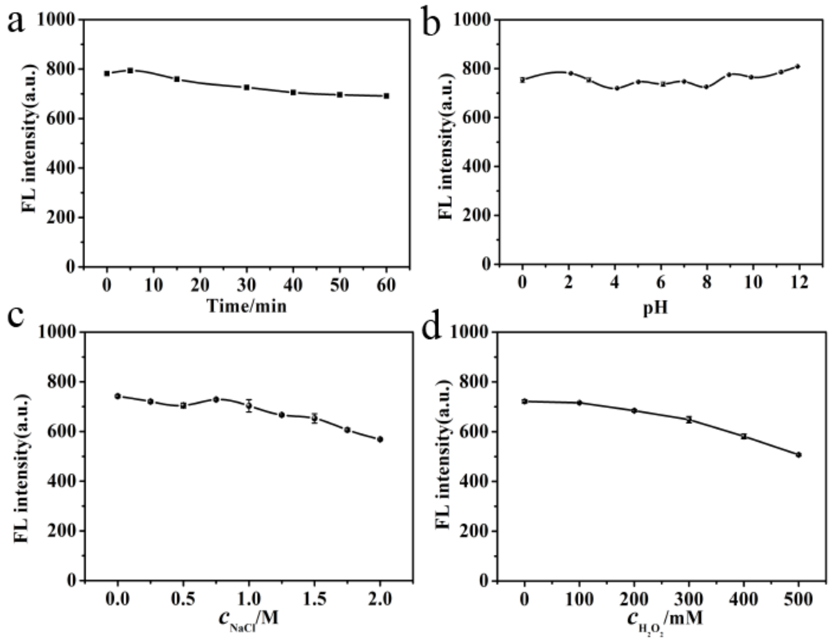

Figure S2. Fluorescent spectra of P-doped CQDs in different incubation time (**a**), pH solutions (**b**) and different concentration of NaCl (**c**), H_2O_2 (**d**).

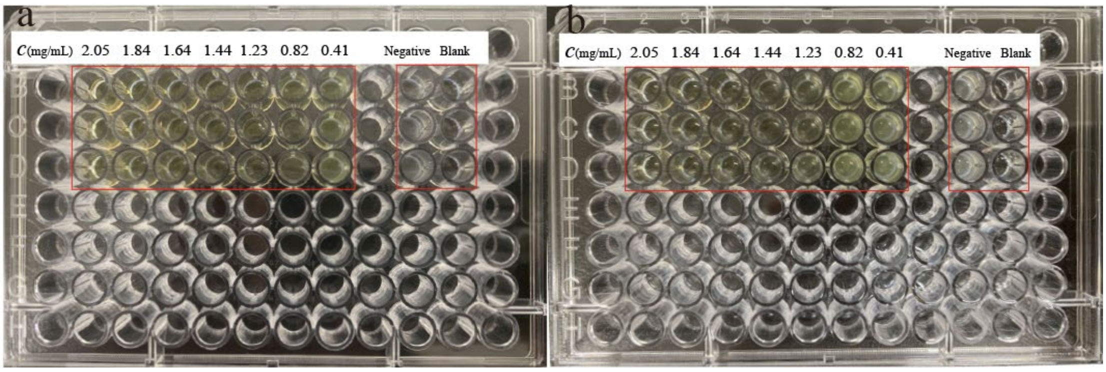

Figure S3. The antibacterial ability of P-doped CQDs on *E. coli* (**a**) and *S. aureus* (**b**).

Table S1. OD values of *E. coli* treated with different concentrations of P-doped CQDs.

c_{CQDs} (mg/mL)	2.05	1.84	1.64	1.44	1.23	0.82	0.41	Negative group	Blank group
	0.116	0.101	0.089	0.084	0.072	0.178	0.226	0.480	0.047
OD	0.110	0.099	0.087	0.085	0.074	0.112	0.261	0.452	0.044
	0.108	0.092	0.092	0.087	0.070	0.139	0.221	0.470	0.045

Table S2. OD values of *S. aureus* treated with different concentrations of P-doped CQDs.

c_{CQDs} (mg/mL)	2.05	1.84	1.64	1.44	1.23	0.82	0.41	Negative group	Blank group
	0.099	0.082	0.070	0.061	0.223	0.489	0.501	0.574	0.043
OD	0.102	0.087	0.063	0.060	0.229	0.447	0.580	0.475	0.041
	0.095	0.088	0.061	0.064	0.202	0.437	0.521	0.614	0.043
